# Stress, coping strategies and expectations among breast cancer survivors in China: a qualitative study

**DOI:** 10.1186/s40359-021-00515-8

**Published:** 2021-02-08

**Authors:** Ruo-Yu Hu, Jing-Ya Wang, Wan-Li Chen, Jie Zhao, Chun-Hai Shao, Ji-Wei Wang, Xiao-Min Wei, Jin-Ming Yu

**Affiliations:** 1 Key Lab of Health Technology Assessment of National Health Commission, School of Public Health, Fudan University, Shanghai, China; 2 Duke Global Health Institute, Duke University, Durham, NC USA; 3 Department of Nutrition, Huashan Hospital, Fudan University, Shanghai, China; 4 Shanghai Health Promotion Center, Shanghai, China

**Keywords:** Breast cancer, Stress, Coping strategies, Qualitative research

## Abstract

**Background:**

Breast cancer is a common tumor in China and has become a public health problem in modern society. Stress plays an important role in the occurrence and progression of cancer. At present, the current situation of stress on breast cancer survivors (BCSs) in China has not been fully understood. This study aims to explore the stress and coping strategies of Chinese BCSs, which provide suggestions to help BCSs reduce stress.

**Methods:**

Sixty-three BCSs from the Shanghai Cancer Rehabilitation Club in China were included in this study and were divided into eight focus groups. These were transcribed verbatim, coded using thematic analysis and analyzed using NVivo 11.

**Results:**

Three themes were extracted from the data to address our research objectives: stress, coping strategies and expectations. The stress of BCSs included psychological stress, stress caused by physical pain, economic stress, stress caused by the change of life status, and stress caused by information overload; the coping strategies included self-strategies and help from others; from the perspective of the survivors, they put forward their expectations for both the society and themselves.

**Conclusions:**

This study shows that BCSs face a variety of stress. In the face of stress, BCSs need comprehensive support, including social and family support to cope with stressors. The findings from this study provide evidence for improving the quality of life among BCSs.

## Background

Breast cancer is one of the three most common cancers in the world, and it is also the most common malignant tumor in Chinese women. The majority of breast cancer cases occur in women aged 35–70 years, and the incidence rates are increasing [1]. By 2021, the incidence of breast cancer in China is expected to rise from less than 60 cases per 100,000 women aged 55–69 years to more than 100 cases per 100,000 women [2]. Advances in medical technology have made remarkable achievements in the early diagnosis and treatment of breast cancer, thereby extending the lifespan of breast cancer survivors (BCSs). At present, breast cancer has become a major public health problem in modern society, and it is managed as a chronic disease [3–5].

BCSs are vulnerable to a variety of stress due to the consequences of cancer diagnosis and treatment. For example, fear of recurrence, financial burden of cancer treatment and perceived discrimination in the social participation and employment can place great stress on BCSs [6]. Moreover, modified radical mastectomy, the most common surgery for breast cancer, causes permanent changes in a woman's appearance [7], which induces re-experiencing in uneasiness of breast changes, and avoiding to body exposure [6, 8, 9]. Additionally, in Chinese culture, cancer is often viewed as "bad luck" or contagious. When BCSs attempt emotional disclosures, they may perceive themselves to be misunderstood, denied, or alienated by their social partners, or even their family members.

According to systematic review, the chronic stress suffered by BCS may not only lead to the deterioration of the disease, but also have a significant negative impact on mental health and quality of life (QoL). The stress leads to many significant adverse outcomes, such as increased risk of depression and suicide, deteriorating social relationships, lower self-esteem, lower libido, decreased sexual satisfaction and decreased neurocognitive [6, 10–15], which are in accordance with the findings from previous studies in China. Appropriate coping strategies are critical in mitigating the adverse effects brought by stress.

Many researches on coping are based on the Stress Appraisal and Coping Model proposed by Lazarus and Folkman [16], in which coping was defined as “constantly changing cognitive and behavioral efforts to manage specific external and/or internal demands that are appraised as taxing and that exceed the resources of the person” [17]. Many studies have investigated coping strategies among BCS. For example, religious faith played a major role in the coping strategies adopted by most Iranian BCSs [18]. Praying, seeking social support, avoiding negative people and having a will to live are the coping strategies among African American BCSs [19]. In a study on BCSs in China, planning, active reconstruction and self-distraction were described as the most common ways of coping [20]. Studies have also shown that the ways of coping have a significant impact on the adaptation to breast cancer and on the progression of the cancer [18–20]. Coping strategies at the stage of breast cancer diagnosis could act as indicators of postoperative psychological adjustments [21]. According to some meta-analytic findings, engaged coping, which aims to manage or reduce stressors, is associated with better mental and physical well-being than disengaged coping, which aims to avoid or ignore stress [22–24].

BCSs may have specific expectations when they perceive stress from a variety of sources. Some studies noted that expectations of earlier returning to work and receiving more social support are important priorities for BCS [25, 26]. Besides, BCSs' expectations for themselves, such as being more optimistic, contribute to their better quality of life. Expectations are closely related to the behavior intention or motivation and are likely to determine the subsequent action. Implementing supportive interventions in the therapeutic, emotional and social domains to meet BCSs’ expectations has been shown to help BCSs adopt positive coping strategies to manage or reduce stress and have a significant protective effect on their health [27].

This study aims to explore the experiences of Chinese BCSs living with breast cancer as well as coping strategies during cancer diagnosis and treatment, and to provide suggestions in helping BCSs reduce stress and improve their quality of life. The rationale of this qualitative design is to scrutinize the living experiences of survivors through their narratives and self-expression, which could gain insights in this area that may not be available if a quantitative method is used.

## Methods

### Design

This qualitative study used focus groups to explore the daily experience of living Chinese BCSs. The interview outline, consisting of semi-structured questions, is presented in the following section.

### Participants

The BCSs were recruited from the Shanghai Cancer Rehabilitation Club (SCRC), which aimed to improve the quality of life of the registered members through psychotherapy, physical training and rehabilitation training. The club had 17 branches and 18,000 registered members in 2019. Around 50,000 BCSs were identified in Shanghai, among which 5,000 registered BCSs in SCRC. The inclusion criteria included: female survivors with a first diagnosis or primary tumor of breast cancer; completion of treatment, surgery, chemotherapy and radiation, in stable condition without currently receiving adjuvant chemotherapy or radiotherapy; able to participate in club activities on their own; with basic reading capability; able to complete the interview and questionnaire on their own; able to express their ideas clearly.

In the existing qualitative researches, 6–8 focus groups are generally included, with 6–8 participants in each group [28, 29]. In this study, 8 focus groups were included with 8 participants in each group. We contacted the 17 branches of the club by phone and email, and selected the first 8 branches that agreed to participate in the study according to the first-come-first-served rule. Each of the eight branches served as one recruitment site for one focus group. Because each of the branches had a WeChat group of registered BCSs, so we distributed the recruitment information containing the inclusion criteria in the WeChat groups firstly. A total of 64 BCSs in the eight WeChat groups volunteered for the study after saw the recruitment information. Then the 64 BCSs were contacted by telephone by the research team with further details of their participation in the study. During the telephone conversation, 4 BCSs felt that the interview was too long, or they were not in Shanghai on the day of the interview, so they refused to participate in the study. We recruited another 4 BCSs using the same way, and finally, 64 BCSs were recruited. However, on the day of the interview, one participant did not come to the site because of some emergency, so a total of 63 BCSs were finally included in this study. The focus group interviews should be held until no new themes emerged [30], and eight focus group interviews were found to be sufficient in this study, so no additional participants were recruited.

### Implementation of focus group interviews

Eight focus group interviews were conducted in May and June 2018, 60–90 min per session. Each focus group interview was conducted in a private room of the club and was facilitated in Mandarin (by W.L.) and, if participants requested, also in Shanghai dialect. The research assistant in focus group interviews (Z.J.) encouraged free-flowing conversation to ensure that all questions had been thoroughly discussed. The participants were also encouraged to introduce any additional topics. All discussions were recorded using a digital voice recorder, and the participants’ nonverbal behaviors and the interviewers’ thoughts on the interviews were also recorded in notes (by J.Y.). Participants’ demographics were obtained from a pre-interview questionnaire. After each focus group’s discussion, each of the participants received a small, culturally appropriate gift (a towel).

### Development of the interview questions

The interview outline used during the interviews was developed by the research team mainly based on the systematic reviews of BCSs mental health outcomes and BCSs psychosocial needs [27, 31, 32]. Parts on experience of cancer, sources of stress, psychological changes, current coping strategies, and needs of BCSs in the reviews and the related literatures were used to construct the list of questions for the interview. Semi-structured focus group interviews were used to optimize participants’ opportunity to express themselves. The interview questions have presented in Additional file 1: Annex 1.

### Transcription and analysis

The analysis was conducted based on the six thematic analysis phases of Braun and Clarke [33], which included: (i) familiarizing yourself with the data, (ii) generating initial codes, (iii) searching for themes, reviewing themes, (v) defining and naming themes and (vi) reproducing the report [15]. This analysis was undertook by Four different authors. First, the recorded audios were transcribed verbatim and double-checked with the hand-written notes (R.Y. and J.Y.). After reading the transcriptions multiple times to promote detailed familiarity with their contents, primary coding was conducted using thematic analysis by NVivo 11. (W.L. and Z.J.). The initial coding summarized “psychological stress”, and codes were gathered together under umbrella codes such as “impact from the mass media”, “self-abasement caused by changes in appearance”, to ease navigation of the hundreds of codes. In the second phase of coding, researchers discussed and made decisions about which codes were the most important and useful by comparing and synthesizing the initial codes. The code were collated to generate potential themes and double-checked with the original data. Three themes were identified in this process and were reviewed by other authors (J.W. and J.M.). Finally, the whole Shanghai project team came together to discuss and decide on the final set of themes, including resolving any discrepancies identified in the initial rounds of thematic analysis. When all the themes, subthemes, and nodes were determined, they were discussed and reviewed by all authors to ensure the accuracy and were used to form the final themes [33]. As it is very essential to guarantee the accuracy of the participants’ quotes when translated from Mandarin to English, a researcher (C.H. and X.M.) who spoke both Mandarin and English checked the translation several times to ensure the greatest possible equivalence between the translation and the original text.

### Ethical approval and informed consent

Ethical approval to conduct this study was granted by the Medical Research Ethics Committee of the School of Public Health, Fudan University. Written informed consent was obtained from each of the participants before the investigation.

## Results

### Demographic characteristics of participants

The participants of this study were recruited from the Shanghai Cancer Rehabilitation Club. A total of 63 voluntarily participating survivors were recruited. The participants were all female BCSs, aged 39–69 years (M = 57.2, standard deviation = 7.3). Demographic and clinical data were collected in the form of questionnaires (Additional file 1: Annex 2).

## Thematic analysis

After coding and summarizing the interviews, three themes and 9 subthemes were extracted: (1) stress: psychological stress, physical pain, economic stress, stress caused by changes in the life status and the stress of information overload; (2) coping strategies: measures taken by the survivors themselves; and external help received by the survivors; (3) expectations: expectations for the society and expectations for themselves. Please see the summary of the themes, subthemes and nodes in Additional file 1: Annex 3.

### Stress

The stress theme included five subthemes: "psychological stress", " physical pain stress ", "economic stress", " stress caused by the changes in life status " and " stress caused by information overload".

- Psychological stress.

Survivors mentioned 5 nodes in psychological stress: " self-abasement", "fear of disease", "the impact of others' experiences", "impact from the mass media" and "worries about the future of family members".

i. Self-abasement

The contents under self-abasement node included "self-abasement caused by the change of appearance"," self-abasement caused by taboo", "alienation from others" and so on. The biggest difference between BCSs and other cancer survivors is that except for breast-conserving surgery, all surgical treatments for breast cancer can cause trauma to the outside shape of the body. The loss of a woman's breast after the surgery can make a huge difference to her appearance. Therefore, most of the BCSs will have a sense of self-abasement due to the change in the appearance.

Now I feel a little strange when I look at myself, I feel like a monster when I see myself in the shower.—1st interview, No.8

In addition to the self-abasement brought by the change in appearance, the more prominent problem was that the BCSs felt self-abased because of their status as "cancer survivors". Because of the fear of being looked at differently or treated unequally by others, survivors chose not to let others know about their illness. Respondents said they were more likely to get along with other patients with the same disease, believing they that had more empathy.

We don’t want others to know about our diseases. If they find out, they'll think you're different from normal people.—1^st^ interview, No.2

No one at my work or in my neighborhood knew I was sick. I don't want it to spread because I feel bad about having this disease.—8th interview, No.5

Nowadays, Chinese people tend to be very scared at the mentioning of cancers. Many survivors were reluctant to let others label them as cancer patients when they learnt about their conditions. At the same time, some respondents worried that others would overstate their illness, which would make survivors more self-abased when they get along with others.

We had a relatively mild cancer, while someone would exaggeratedly say ‘She has cancer. It’s serious.’ 7th interview, No.4

Others said they felt depressed because they were overly concerned by others.

It's just that if you tell someone about your condition and they begin to care about you, you're then in a vulnerable position—5th interview, No.6

The BCSs also believed that a person suffering from the disease might bring bad luck to those in close contact, leading to feelings of self-abasement.

But since I seem to be in such a big place, I keep being afraid of infect others. Although, this disease is not infectious.—5th interview, No.1

In addition to not wanting to let people around them know about their illness, survivors who had the illness and had not left their jobs did not want the organizations where the worked to know about their illness. Except for worrying about how their colleagues would see them, they also worried about the organization in the knowledge of their illness would affect their job.

I don’t want my organization to know about my disease. It’s so embarrassing.—8th interview, No.5

ii. Fear of the disease

Some interviewees said that since being diagnosed with cancer and undergoing the surgery, they often worried about the recurrence or metastasis of tumors that had already been removed. The thought put a lot of psychological stress on them, and they might even be afraid to face the results of the examination.

It’s like having a time bomb in you.—2nd interview, No.8

Any discomfort you feel will implicate you in the disease.—3rd interview, No.1

Metastasis and relapse are common among breast cancer patients. There are lots of cases of metastasis and relapse now. It’s terrified to hear about those cases.—4th interview, No.2

iii. Impact from the Mass Media

The psychological stress of BCSs can be affected by news reports of celebrity who have the same illness. Media coverage of high-profile BCSs can have an impact on the psychological well-being of BCSs.

Beina Yao (famous Chinese singer), who must be very rich and have better doctors, died of breast cancer eventually, making us worry about ourselves since we don’t have as good access to medical help as the celebrities do.—3rd interview, No.4

iv. Impact from Others’ Experience

In the interviews, we found that BCSs' stress was influenced not only by news reports of famous people's illnesses but also by the words and similar experiences of those around them. The progression of others around them had a big impact on them, and the progression of others put a lot of psychological stress on the BCSs. The interviewees said that if they heard that patients around them were getting better, they would also be hopeful about their treatment. But if they heard or saw people around them got worse or died from the same disease, they would immediately wonder if the same tragic outcome would happen to them.

I keep thinking that the same thing would happen to me hearing someone around me have a relapse.—3rd interview, No.4

xxii. Worries about the Family Members.

The BCSs worried that their disease would lead to an increased risk of breast cancer for their children in the future, and even worried that such a family history of the disease would be an obstacle to finding a partner for their children.

I have a daughter. I keep warning my daughter about having a disease like me after I got diagnosed. She should start paying attention to her health now by going to the beauty salon and having massages sometimes, keeping the organs healthy and soft.—6th interview, No.4

Any cancer a parent has had can be stressful for a child, and some families just can't accept it, because they know that the disease is hereditary.—8th interview, No.3

Besides, some interviewees said that because of the uncertainty caused by illness, they tended to imagine and to prepare for their death.

I handed over all the money to my husband and told them what I’m worried about if I died before the surgery. I don't know if I'll ever wake up from the anesthesia—2nd interview, No. 6

- Physical pain stress.

Respondents said they were often depressed by the complications of breast cancer surgery and the physical pain of long-term drug use during recovery.

Breast cancer surgery is often taken to remove the tumor. The surgical wound is large, and the surgical sequelae might have a huge impact on the patients’ daily life.

It looks like we are as normal as others, but one of our hands is nearly crippled and we can’t lift heavy things for the rest of our life.—3rd interview, No.2

The pain and side effects of taking breast cancer drugs, which require long-term adherence, could be unbearable.

I put on more than 10 g because of the endocrine drugs. I used to be in the bike club and I've been working out. Now I had fatty liver and other diseases from weight gain. There are a lot of other diseases that come with this disease.— 3rd interview, No.2

The arms and legs will get swollen because of the drugs and bring so much pain on cold days and rainy days.— 3rd interview, No.4

- Economic Stress.

Contents covered in this topic were "inadequate health care coverage, costly follow-up" and "inability to continue working due to illness, which affects income".

Respondents reported significant financial stress due to the high cost of surgery and subsequent rehabilitation for cancer treatment. The majority of respondents believed that the current proportion of medical insurance reimbursement was low and had short effectiveness, resulting in the survivors themselves to bear the increased financial burden.

My husband is retired, there is not much salary… so I hope the society can help us more. We are financially limited after all, and are very vulnerable people.—4th interview, No.6

Critical illness insurance usually covers for a year and a half to two years, but I have to take five years of medicine. All payment is out of pocket after the expiry of health insurance, and I have to pay half of my retirement salary for the drug.—3rd interview, No.4

Survivors also mentioned that the disease often made it impossible for them to continue working, adding to economic stress.

It feels like my home had collapsed. I lost my labor and I couldn't go to work now. My kids are still young. I’ve worried enough about the financial burden, and then the doctor told me that I needed 300,000 yuan (for treatment). The economic pressure is too much.—2nd interview, No.7

- Stress Caused by Changes in Life Status.

The BCSs said that their lives had changed since their diagnosis, mainly in terms of changes in family life and social life.

During the interview, the interviewees also mentioned that after their diagnosis, in addition to their life situation had changed, it had also brought a lot of impact on their family members, making them feel depressed.

We used to be able to do everything at home, but now we need someone around us whatever we do. I used to be able to carry everything even the gas stoves, but now my hands get swollen whenever I lift something heavy. Now that my husband has retired, he’s always around me and helps me carry heavy things, which is very inconvenient.—4th interview, No.6

Besides, other people's attitudes toward BCSs limited their social activities, which could put stress on the survivors.

I used to be in the art promotion department, but now they won't let me go, saying that I have to take the medicine, go and see a doctor now and then, or might have a sudden attack during the performance. They just won't let us go there anymore.—1st interview, No.8

- Stress Caused by Information Overload.

In the interviews, survivors mentioned that the society was developing rapidly, therefore information channels and sources were extensive, and information was diverse, while meantime publication platforms of authoritative information were lacking. As a result, the general public had doubts about the authenticity of some information and were not sure whether to accept the information or not. For the BCSs, the confusion was caused by the lack of proper dietary guidance and a plethora of treatment options from which they didn't know how to choose.

Sometimes you don't know what kind of doctor to go to. There are occasions when you go to the original surgeon and he says, "don't come to me, the surgery is done.—1st interview, No.2

#### Coping strategies

The BCSs also mentioned some coping strategies for managing and adjusting the stress described above, including the subthemes "how to reduce stress on your own" and "getting help from the outside". " Measures taken by oneself " referred to the measures taken by the survivors themselves to cope with the stress and difficulties brought by the disease; "help from the outside" referred to the help which survivors got through the family, society, friends and other external environments in the course of illness.

- Measures Taken by the Survivors Themselves to Cope with Stress.

The topics of measures to cope with stress included "improving cognition about the disease helps relieve stress", " facing treatment positively helps relieve stress ", "developing hobbies helps relieve stress" and "belief helps relieve stress".

All participants in this study were recruited from the Shanghai Cancer Rehabilitation Club, and the majority of the BCSs reported significant improvements in their mood and stress levels compared to those who did not participate in the club. At the club, survivors could take part in a variety of breast cancer-related seminars, patient meetings, and thereby deepen their knowledge of the disease. They also learn more about the knowledge and advice which could help with the treatment and recovery. In addition to alleviating the fear of the disease and relieving the stress of the survivors, the club also holds regular group activities such as dance classes and film appreciations. Survivors can participate in these activities to enrich their lives and relax at the same time. In the course of participating in the activities, survivors can also communicate with other patients. These series of processes are also very helpful to ease the psychological stress of survivors.

The choir in the cancer club holds activities every Saturday. We sing and dance for two hours every week.—7th interview, No.7

Apart from joining clubs, participants also mentioned many other ways of coping with stress, such as traveling, surfing the Internet, listening to the music, listening to the radio, planting, and talking to others.

I always turn on the radio when I’m in a bad mood and listen to it on my own for a while. I will soon feel better.—7th interview, No.2

I talk to my husband every time I feel stressed, and he will talk me through it. I will also participate in some activities and have fun with others. I feel much better having fun with others.—7th interview, No.9

Others said they found peace of mind and improved mood through faith.

I'll go to the temple and pray to the Buddha. I used to go less, but now I go more. It works.—7th interview, No.4

- Help from the Outside World.

During the interview, the interviewees summarized the help they received after the onset of their illness in three aspects, namely, "family support'', "mutual care among the patients" and "neighborhood and social care". Interviewees said that they had felt the love and care from all walks of life since they became ill. Relatives, friends, neighbors, and others had provided them with a lot of help. Some community organizations had organized support groups to help each other. Some regularly organized group activities for the BCSs to improve their mood, while some community organizations (such as Cancer Rehabilitation Clubs) disseminated knowledge to survivors through lectures, meetings, and other forms. Some non-governmental organizations actively offered condolences and helped cancer survivors in need.

The BCSs said that the help and care of loved ones and friends have helped them relieve stress, reduce self-abasement, relax, move beyond the shadows, as well as move forward with treatment and recovery. Therefore, outside help for BCSs can play a crucial role in coping with stress.

Family members such as my brother and other relatives all came to Shanghai to visit me. They all offer their hands immediately when I’m in need. Family is very important to us.—2nd interview, No.4

Also, the BCS said they felt more connected, more empathetic, and better able to talk to those who were going through the same disease.

Baoshan District is doing great in supporting breast cancer patients. One effort they have made is the club, and the other is that the community leaders often visit and comfort us. They have a monthly event in the auditorium with a lot of people attending it. I think the community and the club have done a really good job.—1st interview, No.4

The interviewees also mentioned that they received a lot of care from the neighborhood committees and the society after the onset of their illness.

The community is still very concerned about us after all. They came every year to console.—6th interview, No.3

We are very unfortunate to have this disease, but the society treats us very well. Everyone cares about us in every aspect, making us feel the warmth from the extended family. We are lucky to have met so many kind-hearted people. The charity foundation also helped me during the last Spring Festival. It makes me feel warm.—2nd interview, No.4

#### Expectations

The topic of expectation mainly included the contents mentioned by the interviewees during the interview except for stress and countermeasures, including two sub-topics of "expectation for the society" and "expectation for themselves", reflecting the respondents’ expectations and recommendations for the government, the society, welfare associations and other organizations in the fight against illness and stress.

- Expectations for the society.

Under the subtheme of "expectation for society", four nodes were included: "expectation for the media", "expectation for anti-cancer organizations", "hope to be treated as normal by people around me", "hope to reform health insurance" and "hope to unify the standard of issuing disability certificate".

The vast majority of the respondents expressed the hope that the media would cover more positive aspects of the anti-cancer campaign and care advice, but less negative news and obscure jargon, so that they could cheer up and optimistically fight the disease.

I don’t think they should report the news of famous people dying of breast cancer, but report more about the supporting activities such as cancer clubs and other inspiring news. We can only get frightened by hearing the bad news. It would be better to broadcast how many years someone has lived after treatment.—3rd interview, No.1

Some BCSs had made suggestions to the cancer rehabilitation clubs, hoping to improve the way they register for joining the club, and hoping that the clubs could organize a variety of recreational activities as well as popular science activities related to post-cancer care.

There are so many members in the cancer rehabilitation club now, and it takes a long queue to register. I don't know how many people are in front of me and I might not be in Shanghai when it’s my turn. It takes a long time to get enrolled.—3rd interview, No.4

Some BCSs said they hope to be treated as normal rather than overly concerned.

I don’t want my family members to treat me as a patient and keep asking me what I want to eat, which is kind of pressure to me and kind of annoying. I hope they can treat me as what they did before I got diagnosed, like a normal person. I can eat whatever they cook. Asking me and caring about me every day will only put more pressure on me instead.—3rd interview, No.4

During the interviews, many survivors also mentioned their expectations regarding the disability certificate. Survivors hoped to be able to unify the criteria for issuing the disability certificate. They believed that their arms were prone to edema, pain, and inability to do heavy work after breast cancer surgery. It looked intact on the outside, but it’s a kind of disability, so they hoped to benefit from the welfare policy for the disabled.

We should have a disability certificate. However, some places admit this but some places do not. There are places where people who operate later have a disability certificate. When I went to apply for it, the staff said we are not eligible for the disability certificate in our district.—3rd interview, No.4

On the other hand, survivors generally reported that the current medical insurance for radical illnesses had short effectiveness and was inconsistent in standards in different areas. They hoped the health care system and medical insurance could be reformed.

It’s still the medical insurance that worries us. We have to pay for the medications by ourselves since they are not covered by the insurance.—3rd interview, No.1

The duration of medical insurance for serious diseases, especially western medicine, is only two years, which is too short. For example, if I take the endocrine medicine, I have to pay more than 1,300 yuan a month, and I have to take other western medicines as well. After the time limit for medical insurance expires, the financial burden is very heavy. Can the time limit of medical insurance be extended a little?—5th interview, No.4

- Expectation for themselves.

During the interview, the interviewees also had great expectations for themselves. The interviewees hoped that in the future they could adjust their mentality to face the disease positively.

Keep yourself in a good mood and try not to think about being sick.—8th interview, No.1

Be happy every day and try not to get too tired.—8th interview, No.5

Some survivors also said they would try to repay their families for their care and to adjust to their best condition.

Just because everyone (the family members) cares about us, we have to repay them. We should not be in a bad mood but try to be happy.—2nd interview, No.5

The BCSs also expressed their desire to pass on their experience of fighting cancer to new patients and to help others.

It's very rewarding to help a new patient, including passing on some experience on things like chemotherapy. For example, I lost my hair during chemotherapy. A previous patient told me to shave my hair and keep the roots in my scalp, otherwise, I would lose all my hair. Later, I passed this on to a later patient and she did what I told her.—2nd interview, No.4

It's time to encourage people who are going through chemotherapy, because we've come a long way from chemotherapy, and it's not easy… I think chemotherapy is very painful.—2nd interview, No.1

## Discussion

Through this study, we focused on BCS to understand the various aspects of stress, coping strategies, as well as their expectations and recommendations for the future. The stress was divided into psychological stress, stress caused by physical pain, economic stress, stress caused by changes in life status, and stress caused by information overload. The survivors' coping strategies were divided into strategies taken by the survivors themselves and help from the outside world. Meantime, the survivors raised expectations for themselves and the society.

Preliminary results show that Chinese BCSs are similar to BCSs in other countries, and will suffer a lot more stress than general cancer survivors [6]. Caucasian BCS indicated that they suffered stress because of physical symptoms of the illness and the side effects of treatment, fear of dying, fear of disease progression and debilitation, the loss of their future, and practical concerns. Compared with Caucasian BCSs, Chinese BCS indicated that they had changes in family life, which lead them anxiety. In Asia, the relationships among family members are interdependent, which is different from situations in Western countries [29]. Besides, other studies revealed that family relationships were the most intimate relationships for Chinese women. According to the findings, family communication problems may cause a lack of initiative to cope with their problems and emotions, which eventually cause stress.

In this study, some Chinese BCSs emphasized that their body changes, fear of relapse, and rejection from others were also the sources of stress. Similar findings were reported in another study that body change can bring stress to the survivors, and it also mentioned BCSs used makeup, hairpiece and breast reconstruction to conceal their physical defects [34]. Therefore, our findings add to the strong evidence of the important role of therapists’ professional psychological counseling. Health care workers need to provide professional guidance for BCSs on how to wear artificial breast to reduce stress about body change [35]. Follow-up care for cancer survivors is very important for helping the patients to accept their illness, and the BCSs will need help and supports from both others and the society [36]. At the same time, the help from medical workers for the rehabilitation of BCSs is also important to mitigate the psychological stress of survivors [37, 38]. Because of public ignorance and misunderstanding toward breast cancer, the BCSs often blame themselves for getting the disease, and they also suffer from segregation and discrimination from others frequently. In this regard, it is the responsibility of the mass media to disseminate cancer knowledge to all through a variety of channels, to guide the public in establishing proper awareness and to dispel the misconception that breast cancer is contagious and cursed, both among the BCSs and among the general population to create a good rehabilitation environment for the survivors [37, 39, 40].

Besides, the participants said that during breast cancer treatment and rehabilitation, there would be serious complications such as side effects of drugs and complications after surgery, and the survivors did not know how to deal with these problems properly, causing a lot of stress on them [41]. Survivors felt that the information provided by health care providers was more reliable and that they did not currently have adequate support from health care providers. Therefore, medical practitioners should provide appropriate rehabilitation guidance for the BCSs, regarding sequelae caused by surgery as well as measures to deal with the side effects of drugs and to reduce the stress caused by physical pain among survivors [42].

Furthermore, the cost of breast cancer treatment and rehabilitation puts enormous financial pressure on the survivors. The government and social welfare organizations shall provide certain financial assistance to the survivors whose families are in extremely difficult economic conditions in response to economic pressure. At the same time, the government should reform the medical insurance, as well as actively promote the establishment and improvement of the medical care system and other social welfare systems, so that the financial burden on the survivors of major diseases such as breast cancer can be reduced. As for the disability certificate, the government should introduce relevant laws and regulations as soon as possible to unify the procedure standards of issuing the disability certificate for BCSs in all provinces and regions. The government should also consider relaxing the restrictions on the procedure of issuing disability certificate for BCSs under reasonable circumstances or enable BCSs to enjoy reasonable preferential treatment under certain conditions by increasing the specific type of disability for BCS and introducing relevant welfare policies.

The disease has led to massive changes in both family and social life of the BCSs. On the one hand, BCS in this study mentioned that despite the inconvenience caused to family members after the onset of the disease and some family disputes, in general, their family members had provided a good supportive environment for them. The care and support from family members play an important role in the recovery of BCSs [43–45], but sometimes the excessive care of others can also cause stress for patients [46]. On the other hand, some leaders or organizations may refuse the patients to participate in group activities or work considering their physical condition. However, work may help them to reintegrate into society, and also have a positive impact on the rehabilitation and mental health of cancer survivors. The government should introduce relevant laws and regulations, as well as develop and evaluate prevention interventions regarding the work problems for the BCSs, enabling the BCSs to have an approach to address their need and right to work [42, 47, 48]. Social support and social interaction are also helpful for the BCSs [37, 49], therefore, the BCSs are encouraged to participate in social activities, interact with their fellow patients, maintain a good mental attitude, establish support groups between old patients and new patients as well as improve psychological support based on religious beliefs [27, 50].

Nowadays, the information is complex since the social information dissemination technologies have developed rapidly. The participants indicated that it’s hard for them to distinguish whether the information they obtained through various methods was true or not. Therefore, it is necessary to set up a medical information consultation service organization to answer the questions arising in the process of rehabilitation of BCSs and provide comprehensive information support for improving the life quality of BCSs [51].

The research we designed considers the dilemma that the BCSs will encounter during the rehabilitation process, which the caregivers, survivors' families and friends often ignore. The induction methods and interview data in this study provide recommendations for improving the future of the BCSs. The data also reflect the views of the BCSs.

Limitations still exist in this study. First, there were practical limits in the choice of study participants. Most of the BCSs enrolled in this study were over 45 years old and had retired before diagnosis. There was a lack of younger interviewees in this study. It is also necessary to analyze and discuss the different types of stress experienced by young survivors as compared to older survivors due to the age of onset for breast cancer tends to be younger in recent years. The results in this study may not be able to be generalized to other populations due to similar backgrounds and ages of the participants. Secondly, there might be some bias in the data on the work status considering the discrepancy among participants in understanding the questionnaire. Third, the focus group interviews might lead to a "collective convergence" phenomenon among opinion leaders in the group, that is, some influential people might influence the direction of discussion, which makes it difficult for other participants to express their different views.

## Conclusion

In this study, we analyzed the stress, coping strategies, and expectations of BCSs in China through the experiences of the BCSs. Survivors suffered from severe physical, psychological, and economic stress. The stress is often reduced through self-regulation and the help of others. The BCSs had raised expectations that the society might help them better recover from the illness. Further research is needed to discuss possible measures to reduce stress among the BCSs, to improve social services and policies, as well as to mitigate stress and improve quality of life among the BCSs.

## Supplementary Information

**Additional file 1: Table 1**. Interview guide. **Table 2**. Summary of basic information of interviewees. **Table 3**. Thematic analysis of interview data.

## Data Availability

The datasets analyzed during the current study are not publicly available because the approval for the sharing of subject data was not obtained for the sharing of subject data from the Medical Research Ethics Committee of the School of Public Health, Fudan University.

## Abbreviation

BCS: Breast cancer survivors
