# Supplementary material for: Stress, coping strategies and expectations among breast cancer survivors in China: a qualitative study
Source: BMC Psychol. 2021 Feb 8;9:26. doi: 10.1186/s40359-021-00515-8 (PMC7869238; doi:10.1186/s40359-021-00515-8)
Supplement: Supplementary file 1 — Additional file 1: Table 1. Interview guide. Table 2. Summary of basic information of interviewees. Table 3. Thematic analysis of interview data. [file 40359_2021_515_MOESM1_ESM.docx]

Annex1: INTERVIEW GUIDE

| Table 1. INTERVIEW GUIDE |
| --- |
| A. What changes do you feel after having breast cancer?  B. What aspects of the stress you're feeling right now?  C. Have you encountered any discrimination or self-abasement in your daily life?  D. How has your relationship with your family changed since your diagnosis?  E. What else do you need?  F. What coping strategies do you use to cope with this kind of stress? |

Annex2 : SUMMARY OF BASIC INFORMATION OF INTERVIEWEES

| Table 2. SUMMARY OF BASIC INFORMATION OF INTERVIEWEES | | |
| --- | --- | --- |
| characteristics | Number of Participants | Proportion(%) |
| Age |  |  |
| 0~45 | 4 | 6.3 |
| 46~59 | 32 | 50.8 |
| 60 and above | 27 | 42.9 |
| Marital status |  |  |
| Legally married | 56 | 88.9 |
| Divorced | 3 | 4 |
| Widowed | 4 | 6.3 |
| Educational |  |  |
| Junior high school or below | 35 | 55.5 |
| Technical school or below | 26 | 41.3 |
| University grade and above | 2 | 3.2 |
| Employment status |  |  |
| Retired | 48 | 76.2 |
| On leave due to illness | 6 | 9.5 |
| Unemployed | 6 | 9.5 |
| Long-term sick leave | 3 | 4.8 |
| Monthly per capita income  (Chinese Yuan) |  |  |
| Up to 3000 | 26 | 41.3 |
| 3001~5000 | 29 | 46.0 |
| 5001 and above | 8 | 12.7 |
| Years since diagnosis |  |  |
| <5 | 58 | 92.1 |
| 5 and more than | 5 | 7.9 |
| Treatment type |  |  |
| Radical mastectomy | 35 | 55.6 |
| Modified radical mastectomy | 6 | 9.5 |
| Simple mastectomy | 16 | 25.4 |
| Partial resection of the breast | 5 | 7.9 |

Annex3: THEMATIC ANALYSIS OF INTERVIEW DATA

| Table 3 THEMATIC ANALYSIS OF INTERVIEW DATA | | |
| --- | --- | --- |
| Theme | Subtheme | Node |
| Stress | Psychological Stress | self-abasement |
|  |  | Fear of disease |
|  |  | Impact from the mass media |
|  |  | Impacts from others’ experience |
|  |  | Worries for family members (especially for daughters) |
|  | Stress caused by physical pain | Sequelae caused by surgical treatment |
|  |  | Side effects |
|  | Economic Stress | Treatment costs too much, and health care coverage is inadequate |
|  |  | Decrease in income due to inability to continue working after being diagnosed with the illness |
|  | Stress caused by changes in life status | Changes in family life |
|  |  | Restrictions on social activities |
|  | Stress caused by information overload | Unable to distinguish between truth and fakeness when there’s so many sources of information |
| Coping strategies | Measures taken by the survivors themselves to cope with stress | Cognitive improvement for the disease helps with stress relief |
|  |  | Facing treatment positively helps relieve stress |
|  |  | Developing hobbies helps relieve stress |
|  |  | Faith helps Relieve Stress |
|  | Help from the outside world | Family support |
|  |  | Communication with other patients |
|  |  | Concern from the Neighborhood Committee and the society |
| Expectations | Expectations for the society | Expectations of mass media |
|  |  | Expectations of the Anti-Cancer organizations |
|  |  | Wish to be treated like a normal person |
|  |  | Hope to unify the criteria for issuing the disability certificates |
|  |  | Hope for the health system reform |
|  | Expectations for the participants themselves | Hope to change the mentality and face the illness with positive attitudes |
|  |  | Wish to repay the family |
|  |  | Wish to help others and pass on their experiences |
